# Comparative efficacy of semiconductor laser and sodium hypochlorite irrigation for elimination of *Enterococcus faecalis* in root canals

**DOI:** 10.3389/fmed.2026.1803478

**Published:** 2026-04-23

**Authors:** Jiamin Lu, Rui Xu, Chen Tang, Min Zhou

**Affiliations:** 1 Department of Endodontics, Changzhou Stomatological Hospital, Changzhou, China; 2 Department of Stomatology, Changzhou Hospital of Traditional Chinese Medicine, Changzhou, China

**Keywords:** antimicrobial efficacy, endodontic treatment, *Enterococcus faecalis*, root canal disinfection, semiconductor laser, sodium hypochlorite

## Abstract

**Background:**

Persistent *Enterococcus faecalis* infection remains a primary cause of endodontic treatment failure, necessitating the development of more effective disinfection protocols. This retrospective case-control study compared the antimicrobial efficacy of semiconductor laser irradiation combined with sodium hypochlorite irrigation vs. sodium hypochlorite irrigation alone for the elimination of *E. faecalis* from infected root canals.

**Methods:**

A total of 102 patients with chronic apical periodontitis and confirmed *E. faecalis* infection who underwent root canal treatment between May 2024 and June 2025 were retrospectively enrolled. Treatment outcomes were evaluated at short-term 6-month follow-up using clinical and radiographic criteria. Patients were divided into two groups: the laser group (*n* = 51), which received 810 nm semiconductor laser irradiation combined with 3% sodium hypochlorite irrigation, and the control group (*n* = 51), which received 3% sodium hypochlorite irrigation alone. Microbiological samples were collected before treatment (S1), immediately after chemomechanical preparation (S2), and after final disinfection (S3). Bacterial counts were determined using colony-forming unit (CFU) analysis, and *E. faecalis* was identified through polymerase chain reaction (PCR). While quantitative real-time PCR (qPCR) offers advantages in specificity and sensitivity, CFU analysis was employed in this retrospective study as it represents the clinically accessible standard method during the study period. Treatment outcomes were evaluated at 6-month follow-up using clinical and radiographic criteria.

**Results:**

Both groups demonstrated significant reductions in bacterial load from S1 to S3 (*p* < 0.001). However, the laser group exhibited significantly greater bacterial reduction rates compared to the control group (98.7 vs. 89.4%, *p* < 0.001). The complete elimination rate of *E. faecalis* at S3 was significantly higher in the laser group (92.2%) compared to the control group (74.5%; *p* = 0.018). At the short-term 6-month follow-up, the laser group showed superior healing rates (90.2 vs. 76.5%, *p* = 0.043). Multivariate logistic regression identified laser treatment (*OR* = 3.42, 95% CI: 1.28–9.15, *p* = 0.014), initial bacterial load (*OR* = 0.67, 95% CI: 0.49–0.91, *p* = 0.011), and tooth type (*OR* = 2.18, 95% CI: 1.05–4.52, *p* = 0.036) as significant predictors of *E. faecalis* elimination.

**Conclusion:**

Semiconductor laser irradiation combined with sodium hypochlorite irrigation demonstrates significantly superior antimicrobial efficacy against *E. faecalis* compared to conventional irrigation alone, resulting in improved short-term clinical outcomes in the treatment of infected root canals. However, as *E. faecalis* represents only one component of the complex endodontic microbiota, future studies employing comprehensive microbiological analysis including qPCR and anaerobic culture are warranted to validate these findings across the polymicrobial infection spectrum. Long-term follow-up studies are warranted to confirm the stability of these results.

## 1 Introduction

Root canal treatment represents the cornerstone of contemporary endodontic therapy, aiming to eliminate microbial infection from the root canal system and prevent subsequent periapical pathology (1). The success of endodontic treatment fundamentally depends upon the effective eradication of microorganisms from the complex root canal anatomy, which harbors diverse bacterial communities capable of establishing persistent infections (2). Despite significant advances in instrumentation techniques, irrigation protocols, and obturation materials, the complete elimination of intracanal bacteria remains an elusive goal, with failure rates ranging from 10 to 30% in primary root canal treatments and substantially higher in retreatment cases (3). This persistent challenge has prompted extensive research into adjunctive disinfection technologies that can enhance the antimicrobial efficacy of conventional chemomechanical preparation.

*Enterococcus faecalis* has emerged as the most frequently isolated microorganism from failed root canal treatments, being identified in 24%−77% of teeth requiring retreatment (4). This gram-positive facultative anaerobe possesses remarkable survival capabilities that enable its persistence in the harsh environment of instrumented and medicated root canals (5). The unique characteristics of *E. faecalis* include its ability to survive in nutrient-depleted environments, tolerate extreme pH levels, resist intracanal medicaments such as calcium hydroxide, and form biofilms that confer additional protection against antimicrobial agents (6). Furthermore, *E. faecalis* can penetrate deeply into dentinal tubules to depths exceeding 1,000 micrometers, where it remains protected from chemical irrigants and systemic immune responses (7). These survival mechanisms collectively render *E. faecalis* particularly resistant to conventional disinfection strategies and establish it as a critical target organism in the development of enhanced root canal disinfection protocols.

Sodium hypochlorite (NaOCl) remains the most widely utilized irrigation solution in root canal treatment, valued for its broad-spectrum antimicrobial activity, tissue-dissolving capability, and cost-effectiveness (8). Concentrations ranging from 0.5 to 6% are routinely employed, with 2.5 to 5.25% representing the most commonly recommended range that balances antimicrobial efficacy with tissue biocompatibility (9). The antimicrobial mechanism of sodium hypochlorite involves the generation of hypochlorous acid and hypochlorite ions, which disrupt microbial cell membranes, denature essential proteins, and oxidize critical enzymatic sites (10). However, despite its established clinical utility, sodium hypochlorite demonstrates significant limitations in eliminating *E. faecalis* biofilms and bacteria residing within dentinal tubules, attributable to the limited penetration depth of chemical irrigants and the protective extracellular matrix of biofilm structures (11). These limitations underscore the necessity for adjunctive disinfection technologies capable of enhancing bacterial elimination beyond what conventional irrigation can achieve.

Laser technology has progressively gained recognition in endodontic practice as a promising adjunctive disinfection modality with the potential to overcome the limitations of conventional chemical irrigation (12). Various laser wavelengths have been investigated for root canal disinfection, including diode lasers, Nd:YAG lasers (1,064 nm), Er:YAG lasers (2,940 nm), and Er, Cr:YSGG lasers (2,780 nm), each possessing distinct tissue interaction properties and antimicrobial mechanisms (13). Among these, semiconductor diode lasers operating at wavelengths have attracted particular attention due to their excellent soft tissue absorption characteristics, compact design, cost-effectiveness, and favorable safety profile (14). The antimicrobial effect of diode lasers derives primarily from photothermal mechanisms, whereby laser energy absorption by bacterial chromophores and surrounding tissues generates localized temperature elevation sufficient to induce protein denaturation, membrane disruption, and cell death (15). Additionally, diode laser energy can penetrate dentinal tubules to depths of approximately 500–1,000 micrometers, potentially reaching bacteria inaccessible to chemical irrigants (1). The semiconductor laser at 810 nm wavelength demonstrates optimal absorption in water and hemoglobin, enabling efficient energy transfer to bacteria while minimizing thermal damage to periradicular tissues (2).

The theoretical framework supporting laser-assisted root canal disinfection encompasses multiple complementary mechanisms that collectively enhance antimicrobial efficacy beyond individual modalities. Photothermolysis represents the primary mechanism, involving direct thermal destruction of bacterial cells through absorption of laser energy by cellular chromophores and subsequent temperature elevation exceeding bacterial survival thresholds (3). Biofilm disruption constitutes a secondary mechanism whereby laser irradiation destabilizes the extracellular polymeric matrix of bacterial biofilms, enhancing the subsequent penetration and efficacy of chemical irrigants (4). Furthermore, the combined application of laser irradiation and sodium hypochlorite may generate synergistic effects through laser-induced agitation of the irrigant, enhanced chemical penetration following biofilm disruption, and potential photochemical activation of hypochlorite ions (5). This multimodal approach targeting bacteria through both physical and chemical mechanisms theoretically provides more comprehensive disinfection than either modality alone.

Despite the promising theoretical basis and encouraging *in vitro* evidence supporting laser-assisted root canal disinfection, significant gaps persist in the clinical literature regarding the efficacy of semiconductor laser irradiation combined with conventional irrigation protocols (6). Previous *in vitro* studies have demonstrated that diode laser irradiation can reduce *E. faecalis* counts by 90 to 99% in infected dentin models, with enhanced efficacy observed when combined with sodium hypochlorite irrigation (7). However, the controlled conditions of laboratory investigations may not accurately reflect the complex clinical environment, where factors including anatomical variations, biofilm maturity, and patient-specific immune responses influence treatment outcomes (8). Clinical studies examining the antimicrobial efficacy of laser-assisted disinfection remain limited in number and methodological rigor, with heterogeneous protocols, small sample sizes, and inconsistent outcome measures precluding definitive conclusions (9). Furthermore, few studies have specifically examined the 810 nm semiconductor laser wavelength, which offers practical advantages over other laser systems in terms of cost, portability, and ease of use (10).

The objective of this retrospective case-control study was to compare the antimicrobial efficacy of 810 nm semiconductor laser irradiation combined with 3% sodium hypochlorite irrigation vs. sodium hypochlorite irrigation alone for the elimination of *E. faecalis* from infected root canals. *E. faecalis* was selected as the target organism due to its well-established role as a key pathogen in persistent endodontic infections and its documented resistance to conventional disinfection protocols (4–7); however, we acknowledge that root canal infections are typically polymicrobial in nature. Additionally, this study aimed to evaluate the short-term clinical outcomes of both disinfection protocols and identify factors predictive of successful *E. faecalis* elimination. We hypothesized that the combined laser and chemical irrigation protocol would demonstrate significantly superior antimicrobial efficacy compared to conventional irrigation alone, resulting in higher *E. faecalis* elimination rates and improved clinical outcomes. The findings of this investigation may contribute to the evidence base guiding clinical decision-making regarding the incorporation of semiconductor laser technology into endodontic disinfection protocols.

## 2 Methodology

### 2.1 Study design and setting

This retrospective case-control study was conducted at the Department of Endodontics, Changzhou Stomatological Hospital, analyzing clinical records and microbiological data from patients who underwent root canal treatment between May 2024 and June 2025. The study protocol received approval from the Institutional Ethics Committee, and the requirement for individual informed consent was waived due to the retrospective nature of the investigation. All procedures were conducted in accordance with the Declaration of Helsinki and institutional guidelines for clinical research.

### 2.2 Participants

Patient records were systematically reviewed to identify individuals meeting the following inclusion criteria: (1) age between 18 and 65 years; (2) diagnosis of apical periodontitis, categorized according to the American Association of Endodontists (AAE) 2009 diagnostic terminology, which includes symptomatic apical periodontitis (characterized by clinical symptoms such as spontaneous pain or percussion sensitivity) and asymptomatic apical periodontitis (characterized by the presence of a periapical radiolucency in the absence of clinical symptoms). All included patients exhibited radiographic evidence of periapical radiolucency. (3) positive culture for *E. faecalis* confirmed by microbiological analysis prior to treatment; (4) single-rooted teeth (incisors, canines, or single-rooted pre-molars) with single root canals; (5) complete clinical and radiographic documentation including pre-treatment, post-treatment, and 6-month follow-up records; (6) no systemic diseases affecting immune function; and (7) no antibiotic use within 30 days prior to treatment.

Exclusion criteria encompassed: (1) teeth with severe periodontal disease (probing depth >5mm); (2) teeth with root resorption, perforation, or fracture; (3) previously root canal-treated teeth; (4) pregnant or lactating women; (5) patients with diabetes mellitus, autoimmune disorders, or immunocompromising conditions; (6) incomplete microbiological sampling data; and (7) patients lost to 6-month follow-up.

Sample size calculation was performed using G^*^Power software (version 3.1.9.7) based on anticipated differences in *E. faecalis* elimination rates between groups. Assuming an elimination rate of 75% in the control group and 92% in the laser group based on preliminary clinical observations and published literature, with α = 0.05 and β = 0.20 (power = 80%), a minimum of 47 patients per group was required. Accounting for potential data incompleteness and follow-up losses, a target enrollment of 50–55 patients per group was established.

### 2.3 Group allocation

Patients were retrospectively assigned to two groups based on the disinfection protocol documented in their clinical records. The laser group (*n* = 51) comprised patients who received 810 nm semiconductor laser irradiation combined with 3% sodium hypochlorite irrigation. The control group (*n* = 51) included patients who received 3% sodium hypochlorite irrigation alone without laser treatment. Group allocation in clinical practice was determined by the treating clinician based on equipment availability and clinical judgment, representing a non-randomized assignment.

### 2.4 Treatment protocol

All root canal treatments were performed by three experienced endodontists (>5 years clinical experience) following standardized clinical protocols. Following local anesthesia administration and rubber dam isolation, access cavities were prepared using high-speed diamond burs with continuous water cooling. Working length was established using electronic apex locator (Root ZX II, J. Morita) and confirmed radiographically, maintaining the apical terminus 0.5–1.0 mm short of the radiographic apex.

Chemomechanical preparation was performed using ProTaper Gold rotary instruments (Dentsply Sirona) in a crown-down sequence, progressing from SX through F3 according to root canal anatomy. Throughout instrumentation, canals were irrigated with 2 ml of 3% sodium hypochlorite solution following each instrument change, delivered using 27-gauge side-vented needles positioned 2 mm short of working length. A total irrigation volume of 20 ml sodium hypochlorite was utilized during preparation.

In the control group, following chemomechanical preparation, a final irrigation sequence was performed consisting of 5 ml of 3% sodium hypochlorite for 60 s, 5 ml of 17% EDTA for 60 s to remove smear layer, and 5 ml of 3% sodium hypochlorite as a final rinse. Canals were dried with sterile paper points prior to obturation.

In the laser group, following identical chemomechanical preparation and preliminary irrigation, semiconductor laser irradiation was delivered using a 810 nm diode laser system (Wiser, Doctor Smile) equipped with a 200 μm flexible fiber optic tip. Laser parameters were set at 1.5 W continuous wave with 15-s irradiation cycles repeated four times, allowing 10-s intervals between cycles for thermal relaxation. The fiber tip was introduced to working length and withdrawn at a rate of approximately 2 mm/s using a helical motion pattern. The root canal was irrigated with 2 ml of 3% sodium hypochlorite between irradiation cycles to provide cooling and remove debris. Following laser treatment, the final irrigation sequence identical to the control group was performed.

All teeth were obturated using cold lateral condensation technique with gutta-percha and AH Plus sealer (Dentsply Sirona) at the same appointment. Coronal access was restored with composite resin following confirmation of adequate obturation radiographically.

### 2.5 Microbiological sampling and analysis

Microbiological samples were collected at three time points: S1 (baseline, before chemomechanical preparation), S2 (immediately after chemomechanical preparation), and S3 (after final disinfection protocol, immediately before obturation). Samples were collected using three sterile paper points (size 25) inserted to working length and maintained for 60 s to absorb residual fluid and dislodge adherent bacteria. Paper points were immediately transferred to sterile Eppendorf tubes containing 1 ml reduced transport fluid and processed within 2 h.

For colony-forming unit (CFU) analysis, samples were vortexed for 30 s, serially diluted (10^−1^ to 10^−6^), and 100 μl aliquots were plated onto Brain Heart Infusion agar supplemented with 5% sheep blood. Plates were incubated aerobically and anaerobically at 37 °C for 48–72 h, and colonies were enumerated. *E. faecalis* identification was performed using conventional biochemical tests (catalase negative, bile esculin positive, growth in 6.5% NaCl) and confirmed by species-specific PCR amplification of the 16S rRNA gene using primers EF1 (5′-ATCAAGTACAGTTAGTCTT-3′) and EF2 (5′-ACGATTCAAAGCTAACTG-3′). PCR products were visualized by gel electrophoresis to confirm the presence or absence of *E. faecalis*.

### 2.6 Outcome measures

The primary outcome measure was the *E. faecalis* elimination rate at S3, defined as the proportion of samples with no detectable *E. faecalis* following final disinfection. Secondary outcome measures included: (1) total bacterial reduction rate, calculated as [(CFU at S1–CFU at S3)/CFU at S1] × 100%; (2) bacterial reduction at each stage (S1 to S2 and S2 to S3); (3) clinical success rate at 6-month follow-up, defined according to the criteria of European Society of Endodontology as absence of clinical symptoms (pain, swelling, sinus tract) combined with radiographic evidence of periapical healing (reduction in radiolucency size or maintained healthy periapical status); and (4) identification of predictive factors for *E. faecalis* elimination.

### 2.7 Data collection

Demographic and clinical data were extracted from patient records including: age, sex, tooth location (anterior vs. pre-molar), pre-operative diagnosis, initial periapical lesion size (measured as greatest diameter on periapical radiograph), duration of symptoms, presence of sinus tract, and treating clinician. Microbiological data included CFU counts at each sampling time point and *E. faecalis* identification results. Follow-up data at 6 months included clinical examination findings and radiographic assessment of periapical status.

### 2.8 Statistical analysis

Statistical analyses were performed using SPSS software version 26.0 (IBM Corp., Armonk, NY, USA) with the significance level set at α = 0.05 for all tests. Continuous variables were assessed for normality using the Shapiro–Wilk test, and normally distributed data were expressed as mean ± standard deviation while non-normally distributed data were presented as median (interquartile range). Categorical variables were expressed as frequencies and percentages. Baseline demographic and clinical characteristics between groups were compared using independent samples *t*-test or Mann–Whitney *U*-test for continuous variables and Chi-square test or Fisher's exact test for categorical variables. Bacterial counts were log10-transformed prior to analysis due to non-normal distribution, and between-group comparisons were performed using Mann–Whitney *U*-test while within-group comparisons across time points used Friedman test with Wilcoxon signed-rank *post-hoc* analysis and Bonferroni correction. *E. faecalis* elimination rates and clinical success rates between groups were compared using Chi-square test with calculation of odds ratios and 95% confidence intervals. Multivariate binary logistic regression analysis was conducted to identify independent predictors of *E. faecalis* elimination, with variables demonstrating *p* < 0.10 in univariate analysis entered into the multivariate model using forward stepwise selection, and model fit was assessed using Hosmer–Lemeshow goodness-of-fit test.

## 3 Results

### 3.1 Demographic and baseline characteristics

**Figure 1:**
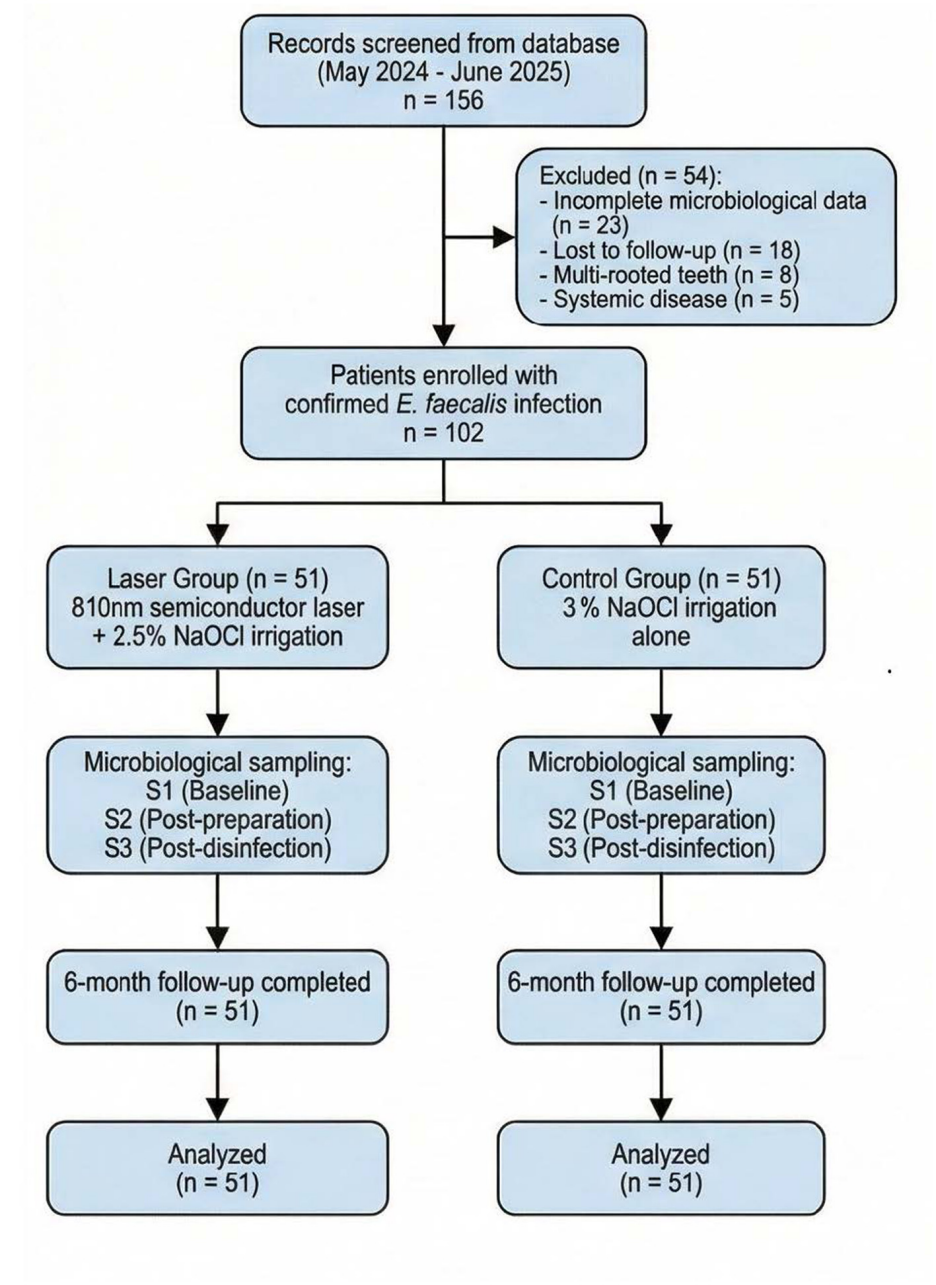
Flowchart of patient selection, group allocation, and study procedures.

A total of 156 patient records were initially screened, of which 54 were excluded due to incomplete microbiological data (*n* = 23), follow-up loss (*n* = 18), multi-rooted teeth (*n* = 8), and systemic disease (*n* = 5). The final sample comprised 102 patients, with 51 in the laser group and 51 in the control group. Demographic and baseline clinical characteristics are presented in Table 1. As shown in Table 1, no statistically significant differences were observed between groups regarding demographic variables or baseline clinical characteristics. The mean age of participants was comparable between the laser group (38.6 ± 11.2 years) and control group (40.3 ± 12.5 years). Sex distribution was relatively balanced, with slightly more females in both groups. Approximately 55%−57% of treated teeth were anterior teeth, with the remainder being single-rooted pre-molars. Baseline bacterial loads, expressed as log10 CFU/ml, were similar between groups (5.84 ± 0.72 vs. 5.91 ± 0.68, *p* = 0.602), confirming comparable infection severity prior to treatment. The patient selection process and study flow are illustrated in Figure 1.

**Table 1:** Demographic and baseline clinical characteristics of study participants.

Variable	Laser group (*n* = 51)	Control group (*n* = 51)	*p*-value
Age (years), mean ± SD	38.6 ± 11.2	40.3 ± 12.5	0.467
Sex, *n* (%)			
Male	23 (45.1)	25 (49.0)	0.685
Female	28 (54.9)	26 (51.0)	
Tooth type, *n* (%)			
Anterior	29 (56.9)	28 (54.9)	0.823
Pre-molar	22 (43.1)	23 (45.1)	
Tooth location, *n* (%)			
Maxillary	31 (60.8)	28 (54.9)	0.541
Mandibular	20 (39.2)	23 (45.1)	
Symptom duration (weeks), median (IQR)	6 (3–12)	8 (4–14)	0.312
Sinus tract present, *n* (%)	12 (23.5)	14 (27.5)	0.651
Periapical lesion size (mm), mean ± SD	4.2 ± 1.8	4.5 ± 2.0	0.423
Baseline CFU/ml (log10), mean ± SD	5.84 ± 0.72	5.91 ± 0.68	0.602

SD, standard deviation; IQR, interquartile range; CFU, colony-forming units.

### 3.2 Bacterial reduction following treatment

**Figure 2:**
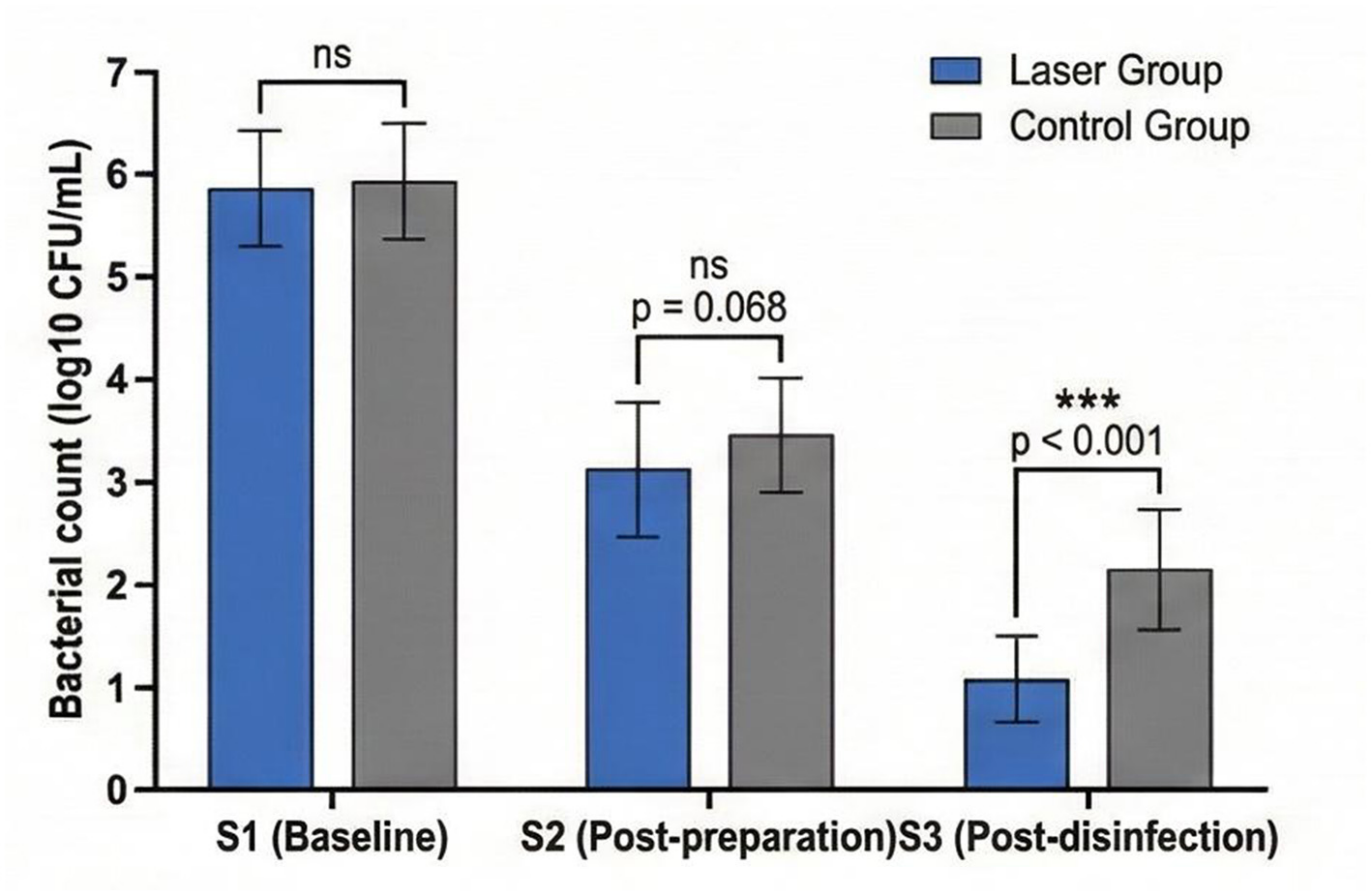
Comparison of log10 bacterial counts (CFU/mL) between laser and control groups at baseline (S1), post-chemomechanical preparation (S2), and post-final disinfection (S3). Error bars represent standard deviation. Error bars represent standard deviation. ns, not significant; ***p < 0.001 between groups.

Bacterial counts at each sampling time point and corresponding reduction rates are presented in Table 2 and illustrated in Figure 2.

**Table 2:** Bacterial counts and reduction rates at each sampling time point.

Parameter	Laser group (*n* = 51)	Control group (*n* = 51)	*p*-value
CFU/ml (log10), mean ±SD			
S1 (Baseline)	5.84 ± 0.72	5.91 ± 0.68	0.602
S2 (Post-preparation)	3.12 ± 0.89	3.45 ± 0.94	0.068
S3 (Post-disinfection)	1.08 ± 0.95	2.14 ± 1.12	< 0.001^*^
Reduction rate (%)			
S1 to S2	95.2 ± 3.8	93.8 ± 4.2	0.083
S2 to S3	89.6 ± 8.4	71.2 ± 12.6	< 0.001^*^
Overall (S1 to S3)	98.7 ± 1.4	89.4 ± 5.6	< 0.001^*^

^*^Statistically significant (p < 0.05).

SD, standard deviation.

The data presented in Table 2 reveal that both treatment protocols achieved significant bacterial reduction from baseline. Chemomechanical preparation alone (S1 to S2) resulted in substantial bacterial load decrease in both groups, with no significant difference observed between the laser group (95.2 ± 3.8%) and control group (93.8 ± 4.2%) at this stage (*p* = 0.083). However, the final disinfection phase (S2 to S3) demonstrated markedly different outcomes between groups. The laser group achieved an additional 89.6 ± 8.4% reduction following laser irradiation combined with final irrigation, whereas the control group achieved only 71.2 ± 12.6% reduction with irrigation alone (*p* < 0.001). Consequently, the overall bacterial reduction rate from S1 to S3 was significantly higher in the laser group (98.7 ± 1.4%) compared to the control group (89.4 ± 5.6%, *p* < 0.001).

As illustrated in Figure 2, final bacterial counts at S3 were significantly lower in the laser group (1.08 ± 0.95 log10 CFU/ml) compared to the control group (2.14 ± 1.12 log10 CFU/ml, *p* < 0.001), representing an approximately 10-fold difference in residual bacterial load between treatment protocols.

### 3.3 *Enterococcus faecalis* elimination rates

**Figure 3:**
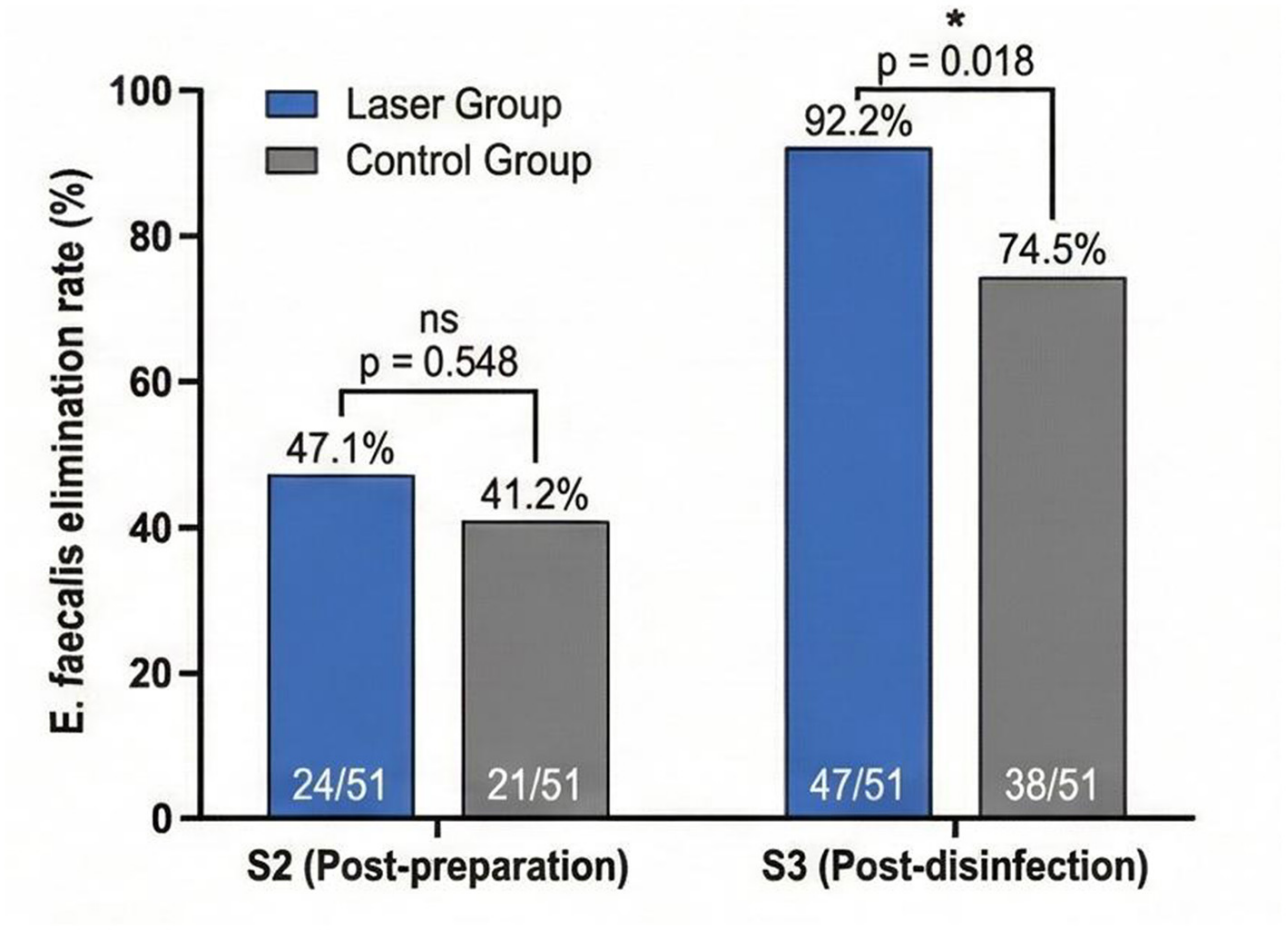
Comparison of *E. faecalis* elimination rates between laser group and control group at post-preparation (S2) and post-final disinfection (S3). ns, not significant. *p = 0.018 between groups at S3.

The *E. faecalis* elimination rates following treatment are presented in Figure 3.

At the S2 sampling point (post-chemomechanical preparation), *E. faecalis* was eliminated in 24 of 51 cases (47.1%) in the laser group and 21 of 51 cases (41.2%) in the control group, with no statistically significant difference between groups (*p* = 0.548). However, following the final disinfection protocol at S3, the laser group demonstrated significantly higher *E. faecalis* elimination, with 47 of 51 cases (92.2%) showing no detectable *E. faecalis* compared to 38 of 51 cases (74.5%) in the control group. This difference was statistically significant (χ^2^ = 5.64, *p* = 0.018), with an odds ratio of 4.02 (95% CI: 1.24–13.05), indicating that patients receiving laser-assisted disinfection had approximately four times higher odds of achieving complete *E. faecalis* elimination compared to those receiving conventional irrigation alone.

### 3.4 Clinical outcomes at 6-month follow-up

**Figure 4:**
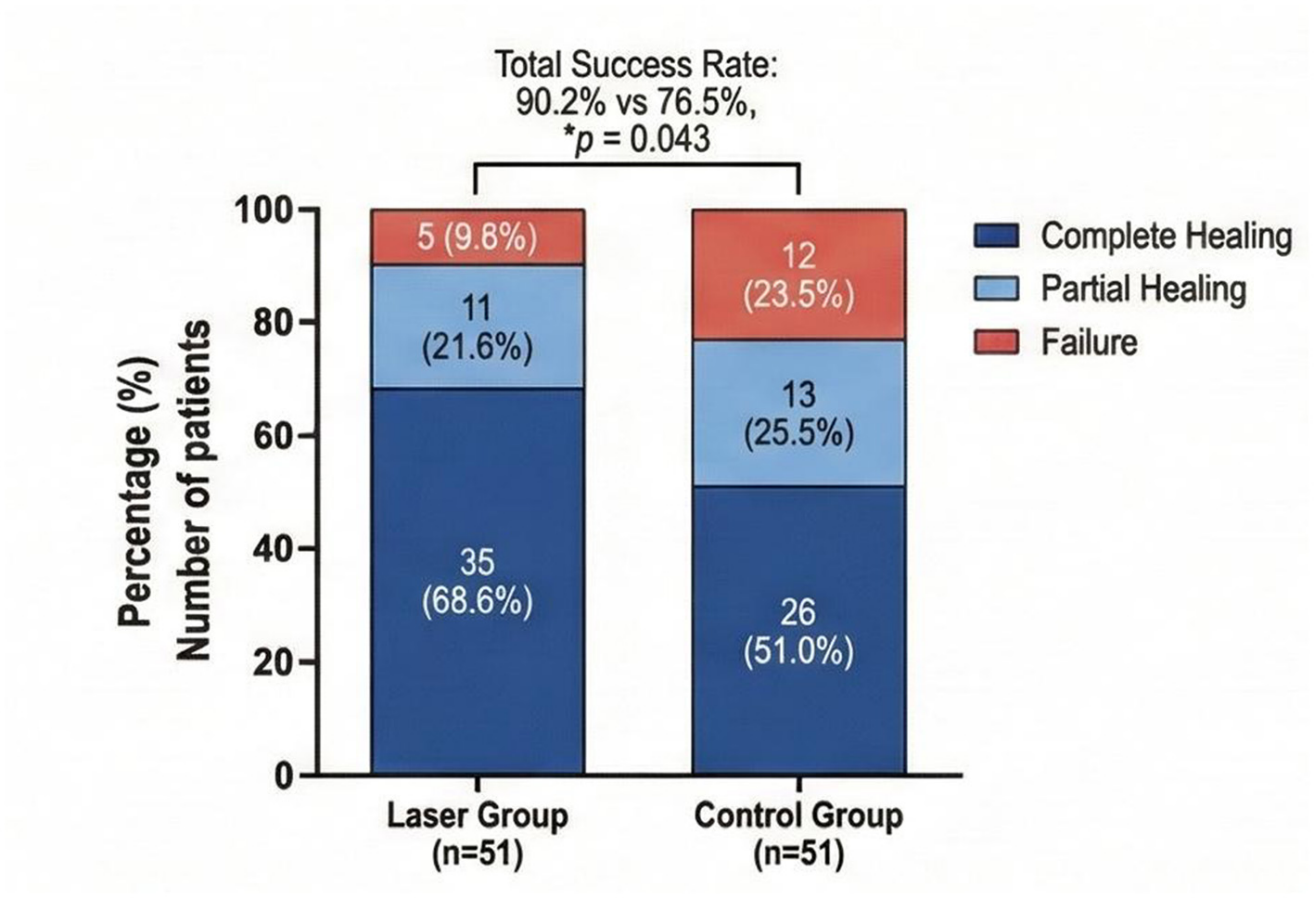
Clinical success rates at 6-month follow-up comparing laser group versus control group, stratified by outcome category (complete healing, partial healing, failure). *p = 0.043 for overall success rate between groups.

Clinical and radiographic outcomes at 6-month follow-up are summarized in Figure 4.

At 6-month follow-up, clinical success (complete or partial healing) was observed in 46 of 51 cases (90.2%) in the laser group compared to 39 of 51 cases (76.5%) in the control group (χ^2^ = 4.09, *p* = 0.043). Complete periapical healing, defined as complete resolution of periapical radiolucency and absence of clinical symptoms, was achieved in 35 cases (68.6%) in the laser group vs. 26 cases (51.0%) in the control group (*p* = 0.068). Partial healing, characterized by reduced lesion size and absence of symptoms, was observed in 11 cases (21.6%) in the laser group and 13 cases (25.5%) in the control group. Treatment failure, defined as persistent or enlarged periapical lesion or presence of clinical symptoms, occurred in 5 cases (9.8%) in the laser group and 12 cases (23.5%) in the control group.

Among cases that failed to achieve *E. faecalis* elimination at S3, treatment failure at 6-month follow-up occurred in 3 of 4 cases (75.0%) in the laser group and 8 of 13 cases (61.5%) in the control group. Conversely, among cases achieving *E. faecalis* elimination, failure occurred in only 2 of 47 cases (4.3%) in the laser group and 4 of 38 cases (10.5%) in the control group, suggesting a strong association between microbiological outcome and clinical success.

### 3.5 Predictors of *E. faecalis* elimination

**Figure 5:**
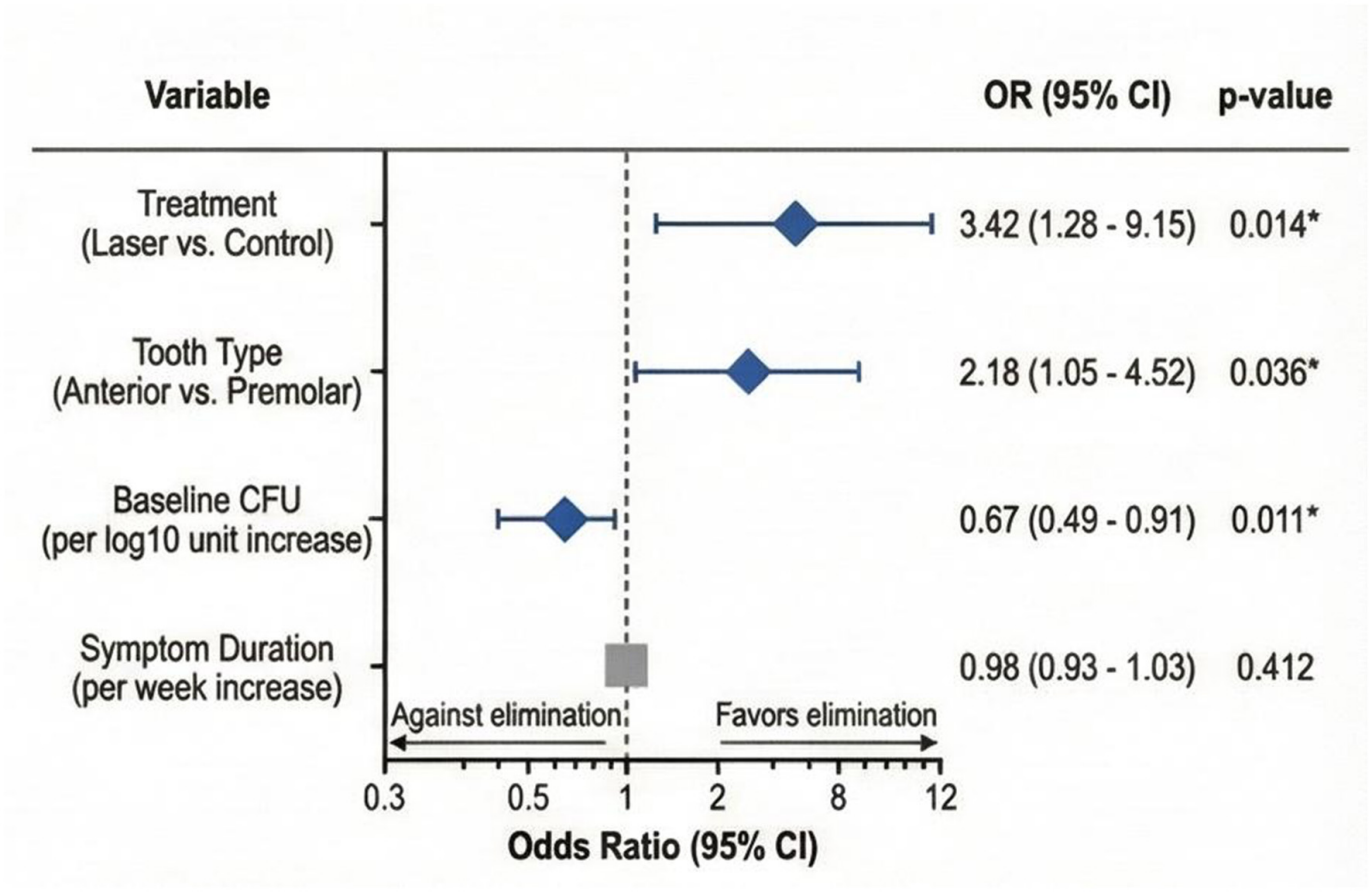
Forest plot displaying odds ratios and 95% confidence intervals for predictors of *E. faecalis* elimination from multivariate logistic regression analysis. *Statistically significant (*p* < 0.05).

Univariate and multivariate logistic regression analyses were performed to identify predictors of *E. faecalis* elimination, with results presented in Table 3 and Figure 5. Multivariate logistic regression analysis, illustrated in Figure 5, identified three independent predictors of successful *E. faecalis* elimination. Laser treatment was the strongest predictor (*OR* = 3.42, 95% CI: 1.28–9.15, *p* = 0.014), indicating that patients receiving laser-assisted disinfection had 3.42 times higher odds of achieving *E. faecalis* elimination compared to those receiving conventional irrigation alone. Anterior tooth location was associated with significantly higher elimination rates compared to premolar teeth (*OR* = 2.18, 95% CI: 1.05–4.52, *p* = 0.036), likely reflecting the simpler canal anatomy of anterior teeth facilitating more thorough disinfection. Initial bacterial load was inversely associated with elimination success (*OR* = 0.67 per log10 unit increase, 95% CI: 0.49–0.91, *p* = 0.011), indicating that higher baseline bacterial counts reduced the likelihood of achieving complete *E. faecalis* elimination. The Hosmer–Lemeshow test indicated adequate model fit (χ^2^ = 6.24, *p* = 0.512).

**Table 3:** Univariate and multivariate logistic regression analysis of factors associated with *E. faecalis* elimination.

Variable	Univariate		Multivariate	
	OR (95% CI)	*p*-value	OR (95% CI)	*p*-value
Treatment (Laser vs. control)	4.02 (1.24–13.05)	0.020	3.42 (1.28–9.15)	0.014^*^
Age (per year increase)	0.98 (0.94–1.02)	0.312	—	—
Sex (Female vs. male)	1.24 (0.52–2.96)	0.628	—	—
Tooth type (Anterior vs. pre-molar)	2.45 (1.02–5.89)	0.045	2.18 (1.05–4.52)	0.036^*^
Baseline CFU (log10, per unit increase)	0.58 (0.35–0.96)	0.034	0.67 (0.49–0.91)	0.011^*^
Lesion size (per mm increase)	0.87 (0.71–1.06)	0.168	—	—
Symptom duration (per week increase)	0.96 (0.91–1.01)	0.089	0.98 (0.93–1.03)	0.412
Sinus tract (Present vs. absent)	0.61 (0.24–1.58)	0.311	—	—

^*^Statistically significant (p < 0.05).

OR, odds ratio; CI, confidence interval; CFU, colony-forming units.

## 4 Discussion

This retrospective case-control study demonstrates that semiconductor laser irradiation at 810 nm combined with sodium hypochlorite irrigation achieves significantly superior antimicrobial efficacy against *Enterococcus faecalis* compared to sodium hypochlorite irrigation alone in the treatment of infected root canals. While previous literature has established the general superiority of laser-assisted disinfection, the present study contributes clinically relevant evidence by specifically evaluating the 810 nm semiconductor laser in a controlled patient population with confirmed *E. faecalis* infection, utilizing standardized clinical protocols and correlating microbiological outcomes with short-term clinical healing. The laser-assisted protocol resulted in higher overall bacterial reduction rates, greater *E. faecalis* elimination rates, and improved short-term clinical outcomes at 6-month follow-up. These findings support the integration of semiconductor laser technology as an adjunctive disinfection modality in endodontic treatment of teeth with persistent *E. faecalis* infection.

The *E. faecalis* elimination rate of 92.2% observed in the laser group represents a substantial improvement over the 74.5% achieved with conventional irrigation alone, corroborating the findings of several previous investigations examining laser-assisted root canal disinfection. Effective elimination of this recalcitrant pathogen constitutes a critical determinant of endodontic treatment success, given the well-established association between persistent *E. faecalis* infection and treatment failure (16). The superior antimicrobial efficacy of the combined protocol likely reflects the complementary mechanisms through which laser irradiation and chemical irrigation target bacteria. While sodium hypochlorite exerts its antimicrobial effect primarily through chemical oxidation of bacterial cell components, laser irradiation induces photothermal destruction of bacteria through direct absorption of laser energy by cellular chromophores (17). The ability of 810 nm laser energy to penetrate into dentinal tubules provides access to bacteria residing in locations beyond the reach of chemical irrigants, addressing a fundamental limitation of conventional disinfection strategies (18).

The magnitude of difference observed between treatment protocols during the final disinfection phase (S2 to S3), where laser irradiation was specifically applied, provides insight into the contribution of laser therapy to overall antimicrobial efficacy. During chemomechanical preparation (S1 to S2), both groups demonstrated comparable bacterial reduction rates of approximately 94%−95%, confirming equivalent baseline preparation quality. However, the additional 89.6% bacterial reduction achieved by laser irradiation during the final disinfection phase significantly exceeded the 71.2% reduction achieved by irrigation alone, representing an approximately 18 percentage point difference attributable to laser treatment (19). This finding suggests that the antimicrobial benefit of semiconductor laser irradiation primarily manifests in eliminating residual bacteria persisting after standard chemomechanical preparation, which represents the most challenging population to eradicate due to their location in protected anatomical niches and biofilm structures (20).

The findings of this study align with and extend previous clinical investigations of diode laser-assisted root canal disinfection. Research conducted by multiple investigative teams has demonstrated that diode laser irradiation at various wavelengths achieves bacterial reduction rates of 85%−99% when combined with conventional irrigation protocols, with *E. faecalis* elimination rates ranging from 78 to 95% depending on study parameters and population characteristics (21). The 92.2% elimination rate observed in our study, while consistent with these reports, provides specific evidence for the 810 nm wavelength in a clinical setting with standardized protocols, addressing a gap in the literature identified by recent systematic reviews (22, 23).

The association between initial bacterial load and *E. faecalis* elimination success identified in multivariate analysis carries important clinical implications for patient management and treatment planning. Cases presenting with higher baseline bacterial counts demonstrated reduced likelihood of achieving complete elimination, suggesting that severely infected teeth may benefit from extended disinfection protocols or multiple laser irradiation sessions (24). This dose-response relationship has been observed in other studies and likely reflects the physical limitation of any disinfection modality to eliminate bacteria exponentially distributed throughout the root canal system and dentinal tubules (25). Clinicians may consider obtaining baseline microbiological cultures in cases of suspected severe infection to guide treatment intensity and establish realistic outcome expectations.

The superior clinical outcomes observed in the laser group at the 6-month follow-up provide preliminary evidence that the enhanced microbiological efficacy may translate into clinically meaningful benefit. The 90.2% clinical success rate achieved with laser-assisted disinfection exceeded the 76.5% success rate observed with conventional irrigation, representing an absolute difference of 13.7 percentage points (26). This magnitude of difference, while clinically meaningful, requires cautious interpretation given the short-term follow-up period and retrospective study design. Periapical healing following root canal treatment is a dynamic process that typically evolves over 12–48 months; lesions classified as partial healing at 6 months may progress to complete resolution, remain stable, or even fail with extended observation (27). Therefore, while the 6-month results are encouraging, they should be considered preliminary indicators of treatment success rather than definitive long-term outcomes. Nevertheless, the strong association observed between microbiological outcome (*E. faecalis* elimination) and clinical outcome (periapical healing) supports the validity of bacterial elimination as a surrogate endpoint predictive of treatment success.

The identification of tooth type as an independent predictor of *E. faecalis* elimination, with anterior teeth demonstrating higher success rates than premolars, reflects the influence of root canal anatomy on treatment outcome. Anterior teeth typically possess single, relatively straight canals with larger diameters that facilitate instrumentation, irrigation, and laser fiber insertion, whereas pre-molars may present anatomical complexities including canal curvatures, isthmi, and accessory canals that harbor bacteria inaccessible to treatment (28). This finding suggests that laser-assisted disinfection provides maximum benefit in anatomically favorable teeth, while complex anatomical configurations may partially attenuate the advantages of adjunctive laser therapy. Future research should examine whether modified laser protocols, including longer irradiation times or multiple fiber insertions, can overcome anatomical limitations in posterior teeth with complex canal systems (29).

The clinical implications of this study support consideration of semiconductor laser irradiation as an adjunctive disinfection modality in specific clinical scenarios. Teeth with confirmed or suspected *E. faecalis* infection, including retreatment cases and teeth with persistent periapical pathology, may derive particular benefit from laser-assisted disinfection given the well-documented resistance of this organism to conventional chemomechanical preparation. The favorable safety profile of semiconductor lasers, combined with their relative affordability and ease of integration into clinical practice, enhances the practical applicability of this technology (30). However, the additional treatment time, equipment costs, and technique sensitivity associated with laser application must be weighed against anticipated benefits in individual case selection.

Several limitations of this study warrant acknowledgment when interpreting the findings. The retrospective case-control design precludes establishment of causal relationships and introduces potential selection bias in group allocation, as clinician preference influenced treatment protocol selection. Although baseline characteristics were statistically comparable between groups, unmeasured confounding variables may have influenced outcomes. The single-center setting and limited sample size restrict generalizability of findings to broader populations and practice settings. A key limitation is the 6-month follow-up period, which, while providing useful short-term outcome data, is insufficient for definitive assessment of long-term treatment success. Periapical healing is a slow process that may require 12–48 months for complete resolution, and the stability of the observed clinical improvement beyond 6 months remains unknown (27). Regarding microbiological assessment, the use of traditional culture-based CFU analysis, while clinically practical and appropriate for this retrospective study, has inherent limitations compared to more advanced molecular techniques such as quantitative real-time PCR (qPCR). qPCR offers enhanced specificity, sensitivity, and the ability to detect non-culturable microorganisms, and future prospective studies should incorporate qPCR for more comprehensive microbial profiling (17, 18). Furthermore, this study focused exclusively on *E. faecalis* as the target organism. While *E. faecalis* is a key pathogen in persistent endodontic infections, root canal infections are typically polymicrobial, involving a diverse array of anaerobic bacteria (1, 2). The antimicrobial efficacy of the tested protocols against the broader endodontic microbiome, particularly obligate anaerobes, remains to be elucidated. Microbiological sampling using paper points, while clinically practical, may underestimate bacterial counts in dentinal tubules and complex anatomical areas. Finally, the non-randomized allocation prevents definitive conclusions regarding the causal effect of laser treatment on observed outcomes.

In conclusion, this retrospective case-control study demonstrates that 810 nm semiconductor laser irradiation combined with sodium hypochlorite irrigation achieves significantly superior antimicrobial efficacy against *Enterococcus faecalis* compared to sodium hypochlorite irrigation alone, resulting in higher bacterial elimination rates and improved short-term clinical outcomes. While acknowledging the limitations of focusing on a single bacterial species and using traditional culture-based methods, these findings provide clinically relevant evidence supporting the consideration of semiconductor laser technology as an adjunctive disinfection modality in endodontic treatment of infected root canals, particularly in cases with confirmed or suspected *E. faecalis* infection. However, given the preliminary nature of the 6-month clinical results and the methodological constraints discussed, prospective randomized controlled trials employing advanced molecular techniques such as qPCR for comprehensive polymicrobial analysis, and with extended follow-up periods (e.g., 2–4 years), are critically warranted to confirm the longevity of these observations, establish definitive clinical recommendations, and fully assess the long-term benefit of integrating laser-assisted disinfection into standard endodontic protocols.

## Data Availability

The raw data supporting the conclusions of this article will be made available by the authors, without undue reservation.

## References

[1] Siqueira JF Jr Rôças IN. Present status and future directions: microbiology of endodontic infections. Int Endod J. (2022) 55:512–30. doi: 10.1111/iej.13677 34958494

[2] Wen Y Luo Y Wei X . Antibacterial effects of liquid discharge cold plasma on *Enterococcus faecalis* planktonic cultures and biofilms: an in vitro study of root canal treatment. J Phys D Appl Phys. (2022) 55:365204. doi: 10.1088/1361-6463/ac7423

[3] Chugal N Mallya SM Kahler B Lin LM. Endodontic treatment outcomes. Dent Clin North Am. (2017) 61:59–80. doi: 10.1016/j.cden.2016.08.009 27912819

[4] Johnsen I Bårdsen A Haug SR. Impact of case difficulty, endodontic mishaps, and instrumentation method on endodontic treatment outcome and quality of life: a four-year follow-up study. J Endod. (2023) 49:382–9. doi: 10.1016/j.joen.2023.01.005 36709041

[5] Jaafar SS. Enterococcus faecalis: a mini-review. J Univ Babylon Pure Appl Sci. (2022) 30:191–200. doi: 10.29196/jubpas.v30i2.4256

[6] Cathro P McCarthy P Hoffmann P Kidd S Zilm P. Enterococcus faecalis V583 cell membrane protein expression to alkaline stress. FEMS Microbiol Lett. (2022) 369:fnac082. doi: 10.1093/femsle/fnac082 36044998 PMC9491840

[7] Ran S He Z Liang J. Survival of *Enterococcus faecalis* during alkaline stress: changes in morphology, ultrastructure, physiochemical properties of the cell wall and specific gene transcripts. Arch Oral Biol. (2013) 58:1667–76. doi: 10.1016/j.archoralbio.2013.08.013 24112733

[8] Siqueira JF Jr Rôças IN. A critical analysis of research methods and experimental models to study the root canal microbiome. Int Endod J. (2022) 55:46–71. doi: 10.1111/iej.13656 34714548

[9] Elfarraj H Lizzi F Bitter K Zaslansky P. Effects of endodontic root canal irrigants on tooth dentin revealed by infrared spectroscopy: a systematic literature review. Dent Mater. (2024) 40:1138–63. doi: 10.1016/j.dental.2024.05.014 38825554

[10] Guivarc'h M Ordioni U Ahmed HMA . Sodium hypochlorite accident: a systematic review. J Endod. (2017) 43:16–24. doi: 10.1016/j.joen.2016.09.023 27986099

[11] Fiorillo LD' Amico C Meto A . Sodium hypochlorite accidents in endodontic practice: clinical evidence and state of the art. J Craniofac Surg. (2024) 35:e636–45. doi: 10.1097/SCS.0000000000010407 39418527

[12] Freire AEN Carrera TMI de Oliveira GJPL Pigossi SC Júnior NVR. Comparison between antimicrobial photodynamic therapy and low-level laser therapy on non-surgical periodontal treatment: a clinical study. Photodiagnosis Photodyn Ther. (2020) 31:101756. doi: 10.1016/j.pdpdt.2020.101756 32302705

[13] Sachelarie L Cristea R Burlui E Hurjui LL. Laser technology in dentistry: from clinical applications to future innovations. Dent J (Basel). (2024) 12:420. doi: 10.3390/dj12120420 39727477 PMC11674728

[14] Malcangi G Patano A Trilli I . Therapeutic and adverse effects of lasers in dentistry: a systematic review. Photonics. (2023) 10:650. doi: 10.3390/photonics10060650

[15] Nunes LP Nunes GP Ferrisse TM . Antimicrobial photodynamic therapy in endodontic reintervention: a systematic review and meta-analysis. Photodiagnosis Photodyn Ther. (2022) 39:103014. doi: 10.1016/j.pdpdt.2022.103014 35840008

[16] Dos Santos GNA Faria-E-Silva AL Ribeiro VL . Is the quality of root canal filling obtained by cone-beam computed tomography associated with periapical lesions? A systematic review and meta-analysis. Clin Oral Investig. (2022) 26:5105–16. doi: 10.1007/s00784-022-04558-y 35618962

[17] Dias LM Ferrisse TM Medeiros KS Cilli EM Pavarina AC. Use of photodynamic therapy associated with antimicrobial peptides for bacterial control: a systematic review and meta-analysis. Int J Mol Sci. (2022) 23:3226. doi: 10.3390/ijms23063226 35328647 PMC8953507

[18] Fiegler-Rudol J Grzech-Leśniak Z Tkaczyk M Grzech-Leśniak K Zawilska A Wiench R. Enhancing root canal disinfection with Er:YAG laser: a systematic review. Dent J (Basel). (2025) 13:101. doi: 10.3390/dj13030101 40136729 PMC11941447

[19] Bordea IR Hanna R Chiniforush N . Evaluation of the outcome of various laser therapy applications in root canal disinfection: a systematic review. Photodiagnosis Photodyn Ther. (2020) 29:101611. doi: 10.1016/j.pdpdt.2019.101611 31809911

[20] Siqueira JF Jr Antunes HS Pérez AR . The apical root canal system of teeth with posttreatment apical periodontitis: correlating microbiologic, tomographic, and histopathologic findings. J Endod. (2020) 46:1195–203. doi: 10.1016/j.joen.2020.05.020 32525058

[21] Siqueira JF Jr Pérez AR Marceliano-Alves MF . What happens to unprepared root canal walls: a correlative analysis using micro-computed tomography and histology/scanning electron microscopy. Int Endod J. (2018) 51:501–8. doi: 10.1111/iej.12753 28196289

[22] Bapat RA Eusufzai SZ Akram Z Chaubal T Phaik KS Seow LL . Antibacterial efficacy of quaternary ammonium compounds (QACs) against *Enterococcus faecalis* in endodontic infections: a systematic review and meta-analysis. BMC Oral Health. (2025) 25:1301. doi: 10.1186/s12903-025-06573-3 40775318 PMC12333173

[23] Dawasaz AA. In vivo efficacy of diode laser as a monotherapy in root canal disinfection: a systematic review and meta-analysis. Photobiomodul Photomed Laser Surg. (2022) 40:59–70. doi: 10.1089/photob.2021.0073 34936823

[24] Asnaashari M Mojahedi SM Asadi Z Azari-Marhabi S Maleki A. A comparison of the antibacterial activity of the two methods of photodynamic therapy (using diode laser 810 nm and LED lamp 630 nm) against *Enterococcus faecalis* in extracted human anterior teeth. Photodiagnosis Photodyn Ther. (2016) 13:233–7. doi: 10.1016/j.pdpdt.2015.07.171 26241781

[25] Lee AHC Cheung GSP Wong MCM. Long-term outcome of primary non-surgical root canal treatment. Clin Oral Investig. (2012) 16:1607–17. doi: 10.1007/s00784-011-0664-2 22205268 PMC3501192

[26] Pinto D Marques A Pereira JF Palma PJ Santos JM. Long-term prognosis of endodontic microsurgery—a systematic review and meta-analysis. Medicina (Kaunas). (2020) 56:447. doi: 10.3390/medicina56090447 32899437 PMC7558840

[27] Ng YL Mann V Gulabivala K. A prospective study of the factors affecting outcomes of nonsurgical root canal treatment: part 1: periapical health. Int Endod J. (2011) 44:583–609. doi: 10.1111/j.1365-2591.2011.01872.x 21366626

[28] Karobari MI Batul R Khan M . Micro computed tomography (Micro-CT) characterization of root and root canal morphology of mandibular first premolars: a systematic review and meta-analysis. BMC Oral Health. (2024) 24:1. doi: 10.1186/s12903-023-03624-5 38167114 PMC10763367

[29] Iandolo A Armogida NG Mancino D Spagnuolo G Cernera M Abdellatif D. Evaluation of root canal cleaning and irrigant penetration using different irrigation protocols: a combined SEM and single-tooth micro-CT study. Clin Exp Dent Res. (2025) 11:e70175. doi: 10.1002/cre2.70175 40674511 PMC12269931

[30] Huang Q Li Z Lyu P Zhou X Fan Y. Current applications and future directions of lasers in endodontics: a narrative review. Bioengineering (Basel). (2023) 10:296. doi: 10.3390/bioengineering10030296 36978686 PMC10044917

